# Head and Neck Cylindroma Masquerading as Squamous Cell Carcinoma: A Case Report

**DOI:** 10.31729/jnma.5067

**Published:** 2020-08-31

**Authors:** Abhilash Konkimalla, Pirabu Sakthivel, Chirom Amit Singh, Rijendra Yogal, Smriti Panda, Mehar Chand Sharma

**Affiliations:** 1 Department of Otorhinolaryngology and Head-Neck Surgery, All India Institute of Medical Sciences, New Delhi, India; 2 Department of Pathology, All India Institute of Medical Sciences, New Delhi, India

**Keywords:** *appendageal tumour*, *cylindroma*, *head and neck tumour*, *histopathology*, *squamous cell carcinoma*

## Abstract

Cylindroma is an uncommon skin appendageal tumor encountered by otorhinolaryngologists in the head and neck. We present a case of a 70-year-old lady who presented with an ulcerative lesion in the pre auricular region. These appendageal tumours can mimic more sinister lesions like squamous cell carcinoma which might warrant overtreatment. This report highlights the importance of harbouring knowledge of these benign tumours in order to provide appropriate management.

## INTRODUCTION

Cylindroma is a rare, benign dermal appendageal tumour which differentiates toward either the eccrine or apocrine line. They occur as solitary or multiple soft, rubbery, skin coloured raised nodular lesions predominantly in the head and neck region. Torso, limbs are rare locations of these tumours (accounting for less than 10% of all cases).^1^ Patients with multiple lesions are very likely to have family members with similar lesions as it has autosomal dominant inheritance pattern.^2^

High degree of clinical suspicion is warranted in these lesions as these may sometimes mimic cutaneous malignancies like adenoid cystic carcinoma, basal cell carcinoma. Correct diagnosis is almost always clinched histopathologically with appropriate immunohistochemistry (IHC).^3^ The nature of lesion and its location makes such patients to visit the dermatologists more often. We present a rare case of solitary cylindroma initially misdiagnosed as cutaneous squamous cell carcinoma which presented in our Otorhinolaryngology and Head-neck surgery department for management.

## CASE REPORT

**Figure 1.**
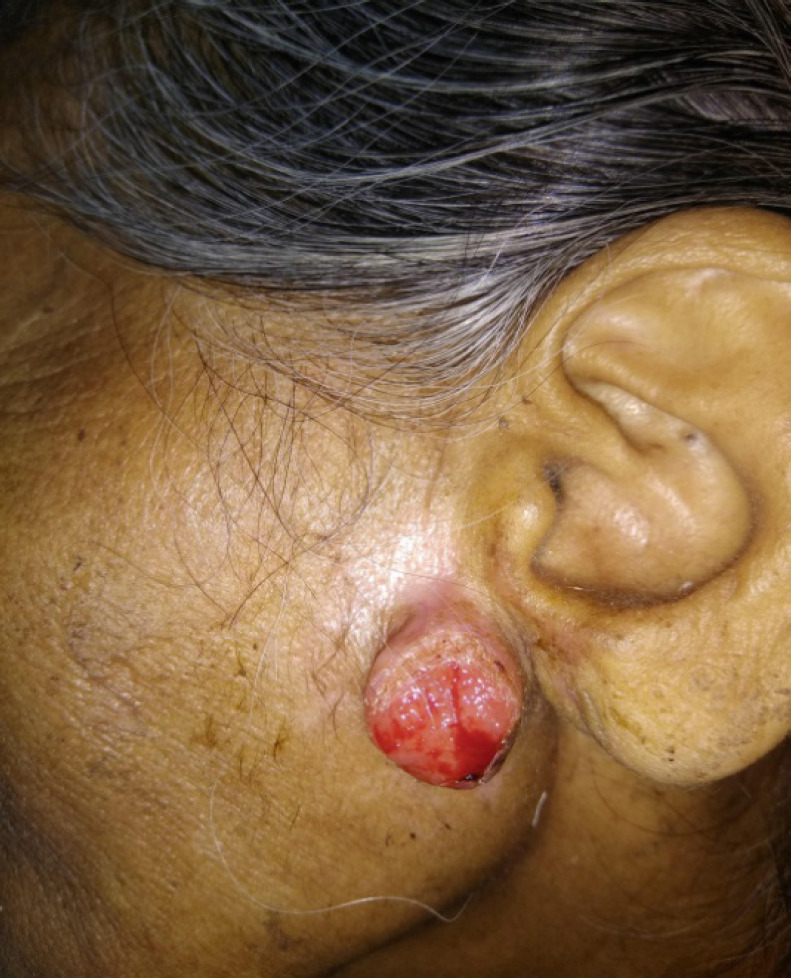
Image depicting a raised nodular lesion in the pre auricular region with superficial skin ulceration mimicking a squamous cell carcinoma.

A 70-year-old female presented with a left sided painless, pre auricular nodular swelling for last 2 years; it was insidious in onset and had gradually progressed in size. There was no family history of similar lesions or no significant past history. Subsequent to a Fine needle aspiration cytology (FNAC) done at another centre, the lesion had superficial skin ulceration and bleeding. The FNAC report was suggestive of squamous cell carcinoma and the patient was referred to our centre for further management. On examination, a 2.5×2 cm raised nodular lesion was seen in the pre auricular region just anterior to the ear lobule and tragus. Superficial skin ulceration with dried blood crusts were seen over the lesion (Fig. 1).

**Figure 2.**
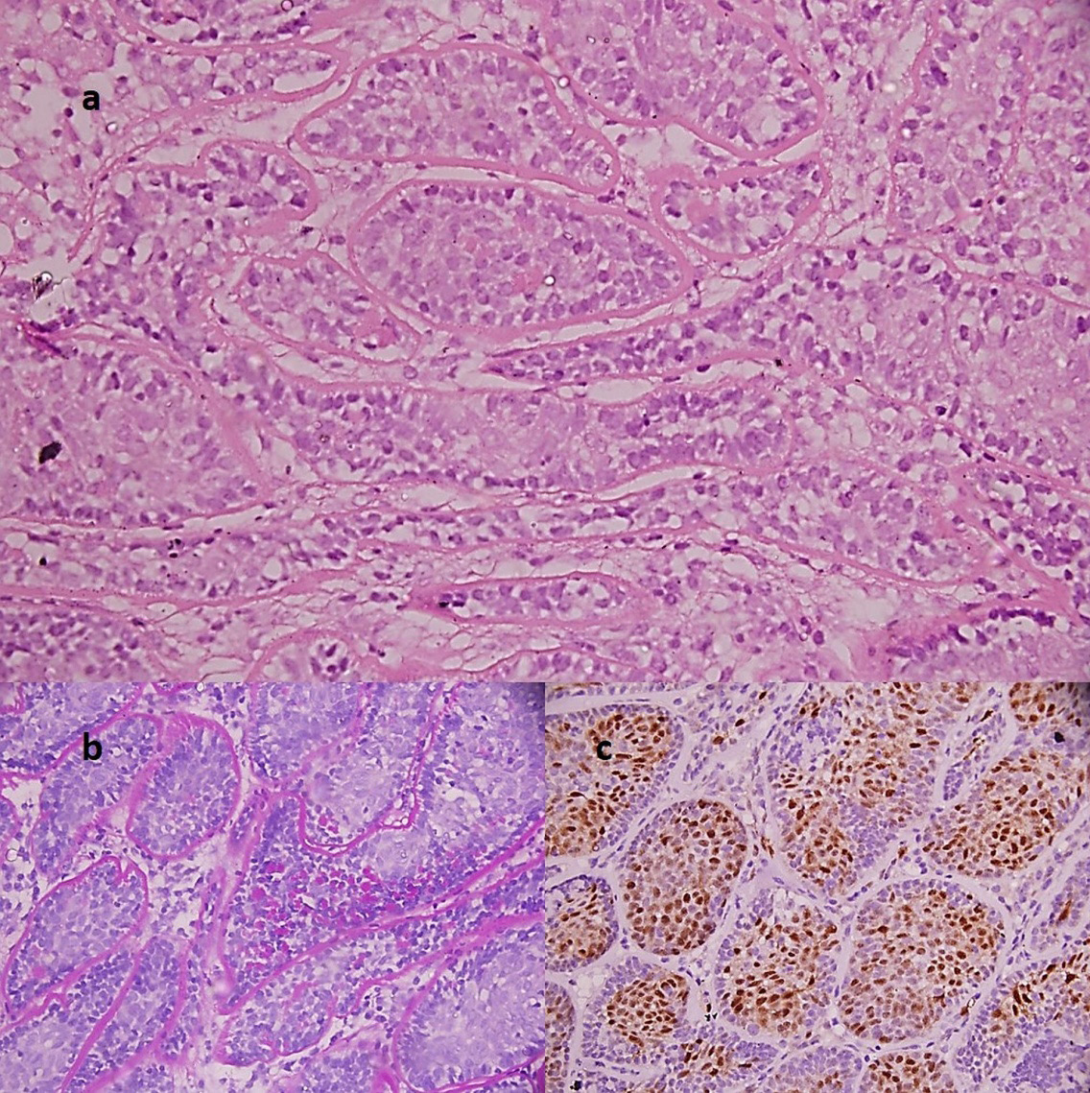
a) Photomicrograph showing islands of tumor cells arranged in a jigsaw puzzle pattern (H&E, ×100); b) The hyaline sheath surrounding tumor islands highlighted by PAS stain. Few hyaline droplets are also noted within the islands (PAS stain, ×400); c) Tumor cells are diffusely immunopositive for cyclin D1 (IHC, ×400).

There were no clinically palpable lymph nodes. Rest of the systemic examination was normal. However, due to the atypical look and low reliability of the cytology report, a repeat incisional biopsy was performed. It showed an intradermal tumour comprising of lobules and islands of cells arranged in jigsaw puzzle pattern (Fig. 2a) with thick PAS positive and diastase resistant surrounding material (Fig. 2b). Lobules showed two types of cells with peripheral palisading and moderate amount of eosinophilic cytoplasm in the centre. The tumour cells were immunopositive for cyclin D1 (Fig. 2c). All the histopathological features were consistent with a diagnosis of cylindroma.

**Figure 3.**
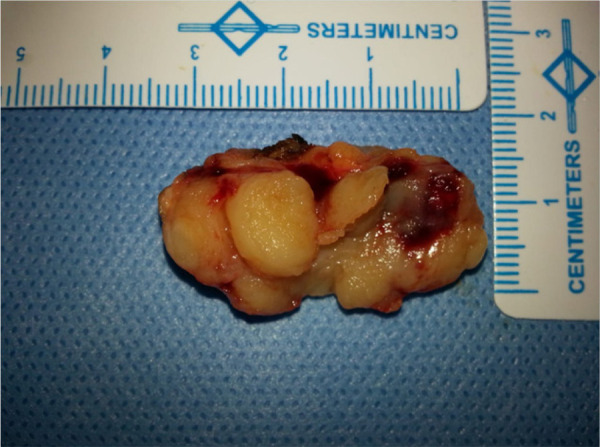
Excision specimen.

The patient underwent complete surgical excision with adequate margins under local anaesthesia. On gross examination, the lesion was exophytic with ulcerated surface. Cut surface was yellowish and solid before formalin fixation (Fig. 3). The final histopathology was consistent with eccrine cylindroma. The patient remained asymptomatic at 12-months follow-up period.

## DISCUSSION

Cylindromas occur in three forms: as a cutaneous lesion like the case we described above; as a lesion in salivary glands which is known as adenoid cystic carcinoma and is malignant; and thirdly as multiple lesion in familial conditions (Brooke Spiegler syndrome), which has a higher malignant potential than cutaneous form. Some of the hereditary conditions like Familial Cylindromatosis, Brooke Spiegler syndrome (BSS) and Multiple Familial trichoepitheliomas were initially considered to be separate entities but recent studies reveal that they are part of the same spectrum of diseases as all involve heterogeneous mutations of the same gene locus, CYLD.^2^

Dermal appendageal benign tumour such as cylindromas can present as lesions which can raise suspicion of a malignant lesion. As head and neck surgeons in India, a superficial ulcerating lesion like in this patient made us ponder about Basal cell/ squamous cell carcinoma rather than the eventual diagnosis. Complete excision of these lesions seems sufficient in the management of these tumours. Given the malignant predilection and high recurrence rates of cylindroma, wide local excision should be considered as first line of management; and the final histopathology post - surgery should direct further line of treatment.^5^ Services of plastic surgeon may be required to reconstruct large facial/ scalp defectspost excision. Electrodessication, cryosurgery and Co2 laser excision are other treatment modalities that have been tried and tested. Malignant transformation is higher in cases of multiple tumours like in BSS and is therefore being emphasised over and over. A multimodality treatment (in form of surgery and radiotherapy) is warranted in such cases.

The origin of these tumours has been a topic of much debate. Sellheyer proposed that both cylindromas and spiradenomas are adnexal neoplasms that were derived from the hair follicle bulge (CD200 positive) and that therefore cylindromas and spiradenomas represent the least differentiated follicular tumours.^6^ On the other hand, cylindromas at a cytomorphological level contain ductal, secretory, eccrine and apocrine glandular components suggesting that these tumours originate from very primitive sweat gland tumours. MYB - NFIB gene fusion was seen in cylindromas as in dermal adenoid cystic carcinoma suggesting similar mechanisms of activation.^7^ Further immunohistochemical and molecular evaluation may put this debate to rest in near future.

Dermatologists are more familiar with this condition than head and neck surgeons or otorhinolaryngologists. But, given the common predilection for head and neck, it is important that divisions dealing with the head and neck like Maxillofacial Surgery, Head and Neck surgery, General surgery be adequately aware about these adnexal masses and their management. This report stresses the need for high clinico-pathological suspicion for avoiding misdiagnoses among head and neck surgeons and pathologists. Long term follow up is required due to high recurrence rate and malignant transformation, especially in multiple tumours.^5^

## Consent:

**JNMA Case Report Consent Form** was signed by the patient and the original article is attached with the patient's chart.

## Conflict of Interest

**None.**

## References

[1] Calonje E (2016). Tumours of Skin Appendages. Rook’s Textbook of Dermatology.

[2] Jordäo C, de Magalhäes TC, Cuzzi T, Ramos-e-Silva M (2015). Cylindroma: an update. Int J Dermatol.

[3] Boggio R (1975). Letter: Adenoid cystic carcinoma of scalp. Arch Dermatol.

[4] Rajan N, Langtry JAA, Ashworth A, Roberts C, Chapman P, Burn J (2009). Tumor mapping in two large multigeneration families with CYLD mutations: Implications for patient management and tumor induction. Arch Dermatol.

[5] Singh A, Ramesh V (2010). Primary cutaneous adenoid cystic carcinoma with distant metastasis: a case report and brief literature review. Indian J Dermatol Venereol Leprol.

[6] Sellheyer K (2015). Spiradenoma and cylindroma originate from the hair follicle bulge and not from the eccrine sweat gland: an immunohistochemical study with CD200 and other stem cell markers. J Cutan Pathol.

[7] Fehr A, Kovàcs A, Loning T, Frierson H, van den Oord J, Stenman G (2011). The MYB-NFIB gene fusion-a novel genetic link between adenoid cystic carcinoma and dermal cylindroma. J Pathol.

